# Prognostic Implications of Exercise-Induced Hypertension in Adults With Repaired Coarctation of Aorta

**DOI:** 10.1161/HYPERTENSIONAHA.122.19735

**Published:** 2022-10-12

**Authors:** Alexander C. Egbe, William R. Miranda, C. Charles Jain, Barry A. Borlaug, Heidi M. Connolly

**Affiliations:** Department of Cardiovascular Medicine, Mayo Clinic Rochester, MN.

**Keywords:** hypertension, exercise, prognosis

## Abstract

The purpose of this study was to assess the relationship between exercise-induced hypertension (EIH) and cardiovascular events, and to determine whether exercise blood pressure (BP) improved risk stratification in adults with repaired coarctation of aorta.

**Methods:**

Retrospective study of patients with repaired coarctation of aorta on antihypertensive therapy that underwent exercise testing and exercise test (2003–2019). BP was measured at rest in 3 different clinical settings and averaged to determine the resting BP. Indices of left ventricular function and afterload were obtained from the echocardiogram. EIH was defined as systolic BP >210 (males) or >190 (females) at peak exercise. Cardiovascular event was defined as atrial fibrillation, ventricular tachycardia, heart failure hospitalization, heart transplant, and cardiovascular death.

**Results:**

Of 327 patients (age 35±13 years), 116 (35%) had EIH. Although the resting BP was similar between patients with versus without EIH, the EIH group had higher pulsatile arterial load, more advanced left ventricular remodeling, and were less likely to be on angiotensin converting enzyme inhibitor/angiotensin receptor blocker. EIH was associated with cardiovascular events (hazard ratio, 1.06 [95% CI, 1.02–1.08]) independent of resting systolic BP, and improved prognostic accuracy above resting systolic BP (*C* statistic, 0.671 [95% CI, 0.645–0.694] to 0.727 [95% CI, 0.709–0.750]; *P*=0.01). Angiotensin-converting enzyme inhibitor/angiotensin receptor blocker was associated with a lower risk of cardiovascular events.

**Conclusions:**

EIH was associated with cardiovascular events independent of resting BP, and patients receiving angiotensin-converting enzyme inhibitor/angiotensin receptor blocker had lower risk of cardiovascular events. These data suggest that exercise BP could be used to assess adequacy of antihypertensive therapy, and to guide titration of antihypertensive therapy.

**Novelty and Relevance**

**What Is New?**

Exercise-induced hypertension was present in 35% of adult with repaired coarctation of aorta treated hypertension and was commonly observed even in the subset of patients with normal blood pressure at rest.

**What Is Relevant?**

Exercise-induced hypertension was associated with cardiovascular events independent of blood pressure at rest. The patients receiving angiotensin-converting enzyme inhibitor/angiotensin receptor blocker were less likely to have exercise-induced hypertension and also had a lower risk of cardiovascular events.

**Clinical/Pathophysiologic Implications?**

Exercise blood pressure can potentially be used to assess adequacy of antihypertensive therapy, and in turn, to guide the titration of antihypertensive therapy

Hypertension is the most common comorbidity in adults with repaired coarctation of aorta (rCOA), and it can persist in up to 40% to 60% of patients after a successful COA repair, even in the absence of hemodynamically significant residual or recurrent coarctation.^1–4^ The diagnosis and monitoring of hypertension in patients with rCOA (without residual or recurrent coarctation) is typically based on blood pressure (BP) measurements obtained at rest (office and/or home BP measurement), similar to the management of hypertension in the general population.^5–7^ However, recent data show that adults with rCOA have higher arterial load as compared with non-COA patients with similar resting systolic BP (SBP), suggesting that resting SBP may underestimate left ventricular (LV) pressure load in this population.^8–10^ The higher arterial load in patients with rCOA is postulated to be due to a combination of endothelial dysfunction, abnormal arterial smooth muscle reactivity, changes in material properties of the aorta leading to increased arterial stiffness, and reduced baroreceptor sensitivity leading to increased sympathetic activation.^11–13^ These same factors are also linked to exercise induced hypertension (EIH).^14–16^

EIH, defined as SBP >210 mmHg in males or >190 mmHg in females, occur in 15% to 30% of patients with rCOA, and it is associated with a higher risk of developing chronic hypertension during follow-up.^17–23^ However, it is unknown whether the presence of EIH in rCOA patients that are already on antihypertensive therapy confers a higher risk of adverse outcomes, independent of BP at rest. Such data could be used to improve risk stratification and optimize antihypertensive therapy in this population. The purpose of this study was, therefore, to assess the relationship between EIH and cardiovascular events both of which are common in this population, and to determine whether exercise BP improved risk stratification in this population.

## Methods

### Study Population

The authors declare that all supporting data are available within the article. The Mayo Clinic Institutional Review Board (IRB) approved this retrospective cohort study of adults (age ≥18 years) with rCOA that were treated for hypertension between January 1, 2003 and December 31, 2019. The IRB number for the Mayo Clinic Adult Congenital Heart Disease Registry was IRB #20-007695, IRB Approval Date: 8/14/2020. The inclusion criteria were the following: (1) clinical diagnosis of hypertension (SBP ≥130 and/or diastolic BP [DBP] ≥80 mmHg) requiring antihypertensive therapy ≥1 year prior to the beginning of the study. (2) Cardiopulmonary exercise test and transthoracic echocardiogram performed within 48 hours from the clinic visit. (3) Clinical follow-up ≥1 year from the time of exercise test for time-to-event analyses. The exclusion criteria were the following: (1) concomitant LV inflow disease (Shone complex) defined as having any of the following conditions: mitral valve prosthesis, sub-valvular, valvular, or supra-valvular mitral stenosis (mean gradient >3 mmHg) or ≥moderate mitral regurgitation. (2) Hemodynamically significant residual COA defined as Doppler mean COA gradient >20 mmHg or need for a transcatheter or surgical COA intervention from the exercise test to the last encounter. (3) Aberrant origin of the right subclavian artery. Figure S1 shows a flowchart for cohort selection.

### Data Acquisition

#### Resting BP

The office BP was measured in 3 different clinical settings: (1) at the time of echocardiogram using a manual BP cuff, (2) at the time of clinic visit using an automated BP cuff, and (3) at the time of exercise test using a manual BP cuff. The BP was measured by trained clinical personnel after 5 minutes of rest in a seated position in all cases. For this study, we used the BP obtained from the right arm, and we averaged the BP measurements from the 3 different clinical settings to determine the average resting BP for each patient. We defined optimal BP control as resting SBP <130 mmHg and resting DBP <80 mmHg.

#### Exercise BP

All patients included in the study underwent maximum effort cardiopulmonary exercise test with a treadmill ergometer. Maximal effort was defined as symptom-limited exercise test with a respiratory exchange ratio >1.10 previously described.^8,17^ BP was measured manually at rest and then every 2 minutes throughout the exercise test. EIH was defined as SBP at peak exercise >210 mmHg in males or >190 mmHg in females.

#### Pulsatile Arterial Load

Pulsatile arterial load was assessed using 2 indices: (1) Effective arterial elastance index, which is a lumped measure of the total stiffness of the arterial system, was calculated as 0.9×brachial systolic BP/Doppler-derived stroke volume index.^9^ (2) Total arterial compliance index, which is a linear approximation of the pressure-volume relationship of the arterial system, was calculated as Doppler-derived stroke volume index/brachial pulse pressure.^9^

#### Echocardiogram

All patients underwent comprehensive 2-dimensional, Doppler and speckle tracking echocardiogram according to contemporary guidelines.^24,25^ The severity of residual COA and LV outflow disease was assessed using standard Doppler techniques. Based on these data, the patients were classified into 2 mutually exclusive groups: (1) Isolated rCOA; (2) rCOA with concomitant LV outflow disease defined as having any of the following conditions: aortic valve prosthesis, sub-valvular, valvular, or supra-valvular aortic stenosis (mean gradient >20 mmHg) or ≥moderate aortic regurgitation.

#### Outcomes

The composite study outcome included incident cardiovascular event defined as new-onset atrial fibrillation, sustained or non-sustained ventricular tachycardia, heart failure hospitalization, heart transplant, and cardiovascular death. Cardiovascular death was defined as death due to myocardial infarction, sudden cardiac death, heart failure, stroke, and cardiovascular hemorrhage.^26^ The occurrence of cardiovascular event was ascertained by review of the electronic health records and the Accurint mortality database.

Additionally, the medical records were reviewed at the time of exercise test, and the clinical indices (including antihypertensive therapy) obtained within 3 months of the exercise tests were used to define the baseline characteristics of the cohort. The medical records were also reviewed for all subsequent clinical encounters to determine intensification of antihypertensive therapy defined as increase in dose of antihypertensive medication and/or addition of another antihypertensive medication.

### Statistical Analysis

Data were presented as mean±SDS, median (interquartile range), and count (%). Between-group comparisons were performed using χ^2^ test for categorical variables, and unpaired *t* test and Wilcoxon rank sum test for continuous variables. Pearson correlation was used to assess the relationship between continuous variables. Annualized cardiovascular event rates were calculated by dividing the number of patients that experienced the event by patient-years of follow-up.

Cox proportional hazard models were used to evaluate the association between exercise SBP and cardiovascular events. First, we created a base model using the following covariates: resting BP, demographic indices (age, sex, body mass index), comorbidities (diabetes, coronary artery disease, atrial fibrillation, renal dysfunction), antihypertensive therapy (modeled as categorical variables), and echocardiographic indices (LV global longitudinal strain, LV mass index, LV filling pressure as measured by E/e’, right ventricular global longitudinal strain, aortic and COA mean gradient). These covariates were chosen using stepwise backwards selection of covariates with *P*<0.1 on univariable analysis.

To assess the prognostic power of exercise SBP over the base model, we added exercise SBP to the base model, and *C*-statistics and the integrated discrimination index were calculated to assess improvement in prediction accuracy.^27^ The integrated discrimination index (95% CI) and comparison *P* were derived from 1000 bootstrap samples. Exploratory analysis was performed to determine whether other exercise BP indices (exercise DBP and exercise pulse pressure [PP]) improved prediction accuracy above the base model.

Sensitivity analysis was performed to assess the relationship between exercise SBP and cardiovascular events in different subsets of patients: (1) males versus females; (2) patients with concomitant LV outflow disease versus isolated rCOA; (3) patients with intensification of antihypertensive therapy after exercise test versus patients that remained in the same antihypertensive therapy. All statistical analyses were performed with SAS version 9.4, and BlueSky Statistics software (version. 7.10; BlueSky Statistics LLC, Chicago, IL), and *P*<0.05 was considered to be statistically significant for all analyses.

## Results

### Baseline Characteristics

There were 327 patients that met the study inclusion criteria. The mean age at baseline assessment was 35±13 years, 201 (62%) were males, and 116 (35%) had EIH. Compared with the patients without EIH, those with EIH had higher LV mass index and LV filling pressures, lower absolute LV global longitudinal strain, and were less likely to be on angiotensin-converting enzyme inhibitor/angiotensin receptor blocker (ACEI/ARB) or multiple antihypertensive medications (Table 1).

**Table 1.**
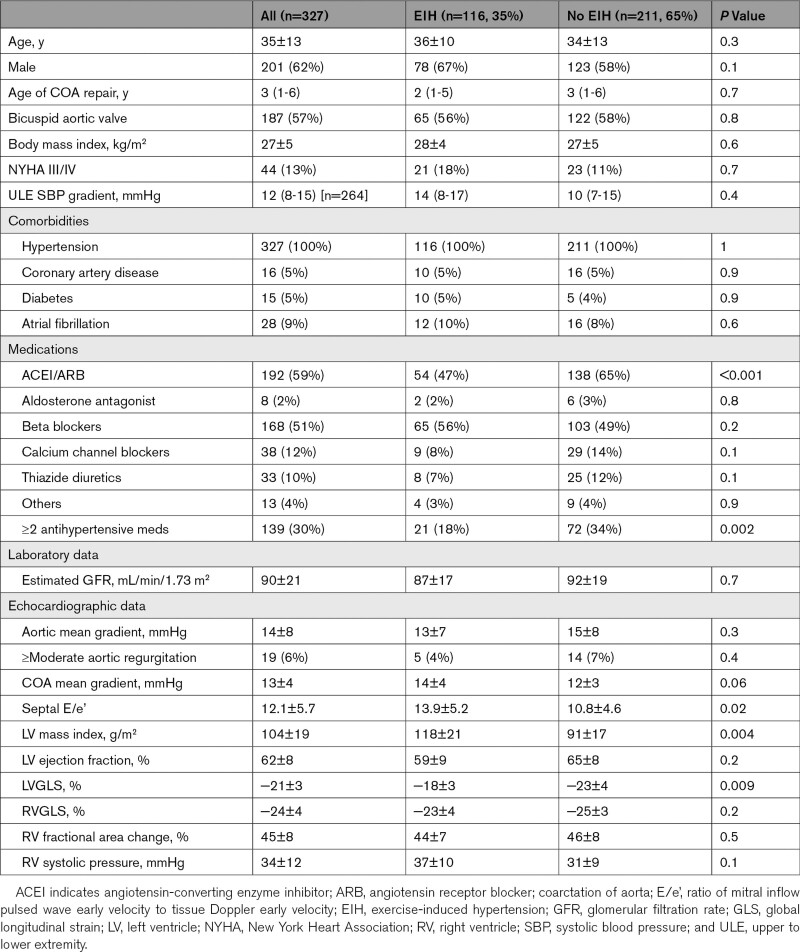
Baseline Characteristics

Of the 327 patients, 215 (66%) had isolated rCOA while 112 (34%) had concomitant LV outflow disease. Table S1 shows a comparison of the baseline characteristics, echocardiographic, and exercise indices between the patients with isolated rCOA versus rCOA with concomitant LV outflow disease. Compared with the isolated rCOA group, those with concomitant LV outflow disease had higher presence of atrial fibrillation, functional limitation, and use of beta blockers, and more advanced LV remodeling (lower absolute LV global longitudinal strain, and higher LV mass and E/e’; Table S1).

### Antihypertensive Therapy

The most common antihypertensive medications were ACEI/ARB (n=192, 59%), and beta blockers (n=168, 51%), and 139 (30%) patients were on 2 or more antihypertensive medications at baseline. Additionally, 89 (27%) patients had intensification of antihypertensive therapy during follow-up (increase in dose n=47, addition of a new medication n=33, increase in dose and addition of a new medication n=9) after undergoing exercise test. Of the 41 patients with initiation of additional antihypertensive medication, the most common agent was ACEI/ARB (n=34), followed by calcium channel blocker (n=5).

### Resting BP

The resting BP measurements in all 3 clinical settings (outpatient clinic, echocardiography laboratory, and exercise laboratory) were obtained on the same day in 178 (54%), and within 2 days in 149 (46%).There was an excellent correlation between BP obtained in the clinic and echocardiography laboratory (SBP: r=0.84; DBP: r=0.65, and PP: r=0.69, *P*<0.001 for all), and between BP obtained in the clinic and exercise laboratory (SBP: r=0.78; DBP: r=0.68, and PP: r=0.71, *P*<0.001 for all). The average SBP was 129±18, and 146 (45%) had optimal SBP control (defined as SBP <130 mmHg). The average DBP was 74±11, and 273 (84%) had optimal DBP control (defined as DBP <80 mmHg). Although, both groups had similar resting SBP, DBP, and PP, the EIH group had higher effective arterial elastance index (3.6±0.7 versus 3.1±0.5, mmHg/mL*m^2^, *P*=0.002), and lower TACI (0.6±0.3 versus 0.9±0.4, mL/mmHg*m^2^; *P*<0.001), suggesting a higher pulsative arterial load and vascular dysfunction in this group (Table 2). There was no difference in the resting BP between patients with isolated rCOA and COA with concomitant LV outflow disease (Table S1).

**Table 2.**
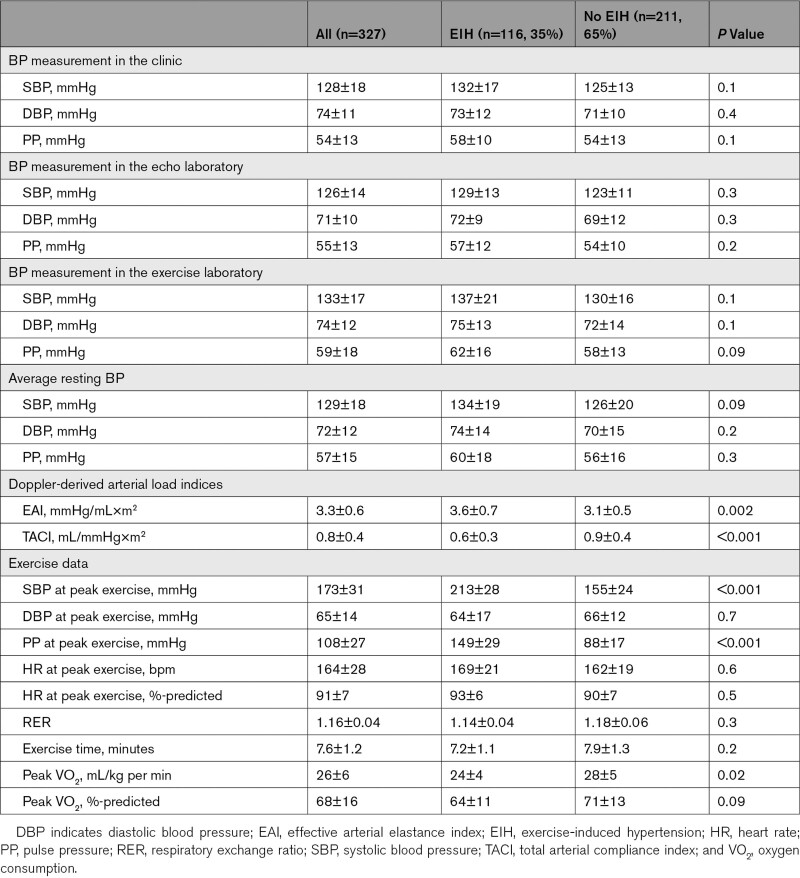
Blood Pressure Data

### Exercise BP

The mean exercise SBP, DBP and PP was 173±31, 65±14, and 108±27 mmHg, respectively (Table 2). There was a poor correlation between resting versus exercise SBP (r=0.31; *P*=0.03), no correlation between resting versus exercise DBP (r=0.14; *P*=0.2), and no correlation between resting versus exercise PP (r=0.29; *P*=0.07). The exercise SBP (by design) and PP was higher in the EIH although both groups had similar effort (as measured by exercise time and respiratory exchange ratio), and peak heart rate (Table 2). There was no difference in the exercise BP between patients with isolated rCOA and COA with concomitant LV outflow disease (Table S1).

### Outcomes

The patients were followed for 7.6±3.2 years from the time of exercise test and during this period, 89 (27%) patients had at least one cardiovascular event (atrial fibrillation n=23, non-sustained ventricular tachycardia n=19, sustained ventricular tachycardia n=6, heart failure hospitalization n=38, and cardiovascular death n=32). The unadjusted incidence of cardiovascular events was 36 per 1000 patient-years. The unadjusted annual incidence of cardiovascular events was lower in patients with optimal resting SBP control as compared with those with suboptimal resting SBP control (32 versus 41 per 1000 patient-years; *P*=0.008; Figure [A]). However, there was no significant difference in cardiovascular event rate between the patients with optimal resting DBP control as compared with those with suboptimal resting DBP control (34 versus 37 per 1000 patient-years; *P*=0.3). Table 3 shows the base model. The resting SBP was independently associated with cardiovascular events (hazard ratio, 1.04 [95% CI, 1.02–1.06]; *P*=0.02). The use of ACEI/ARB was associated with a lower risk of cardiovascular events (hazard ratio, 0.95 [95% CI, 0.92–0.98]; *P*=0.02).

**Table 3.**
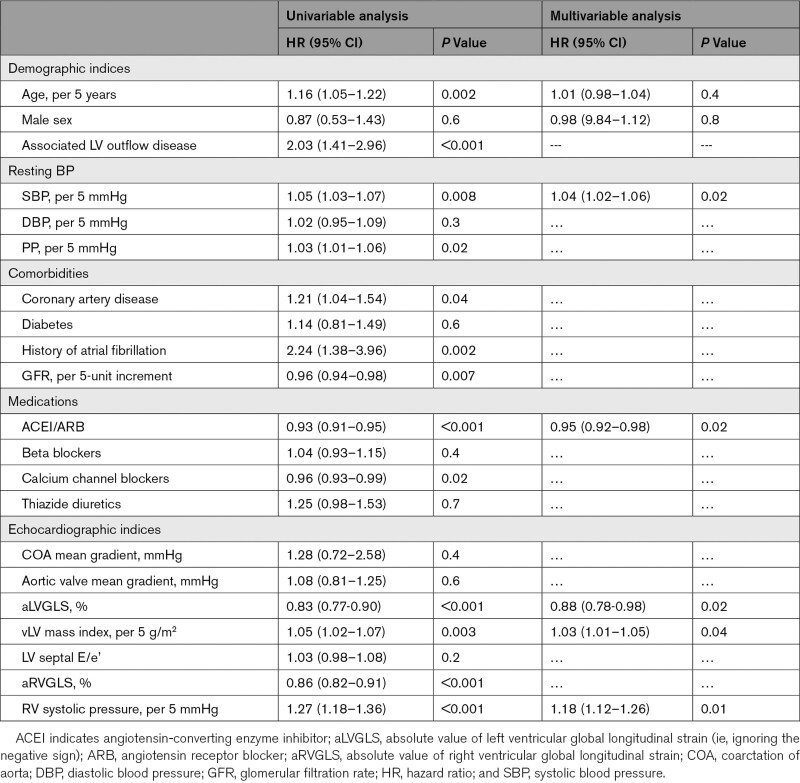
Cox Model Showing Correlates of Cardiovascular Events

**Figure.**
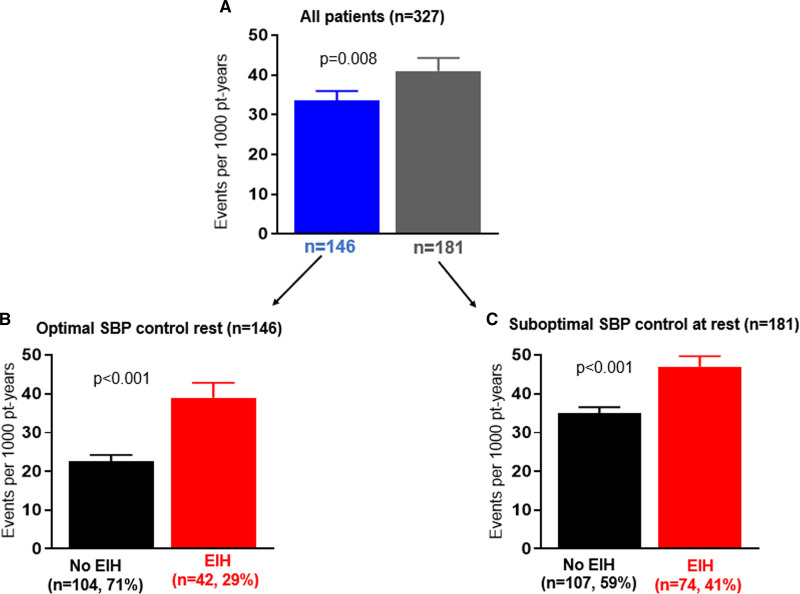
**Bar graphs showing the incidence of cardiovascular event expressed as events per 1000 patient-years. A**, Comparison of events rates between patients with optimal systolic blood pressure (SBP) control at rest (blue) versus patients with suboptimal SBP control at rest (gray). **B**, Subgroup analysis restricted to patients with optimal SBP at rest (n=146). In this subgroup of patients, those with exercise-induced hypertension (EIH; red) had a higher incidence of cardiovascular events. **C**, Subgroup analysis restricted to patients with suboptimal SBP at rest (n=181). In this subgroup of patients, those with EIH (red) had a higher incidence of cardiovascular events.

### Exercise BP and Prognostication

Of the 146 patients with optimal SBP control at rest, 42 (29%) had EIH, and the incidence of cardiovascular events was higher in patients with EIH as compared with patients without EIH (36 versus 23 per 1000 patient-years; *P*<0.001; Figure [B]). Of the 181 patients with suboptimal SBP control at rest, 74 (41%) had EIH, and the incidence of cardiovascular events was also higher in patients with EIH as compared with patients without EIH (47 versus 35 per 1000 patient-years; *P*<0.001; Figure [C]).

Exercise SBP was independently associated with cardiovascular events after adjustment for resting SBP, demographic indices, comorbidities, antihypertensive medications, and echocardiographic indices (hazard ratio, 1.06 [95% CI, 1.02–1.08] per 5 mmHg increase in exercise SBP, *P*=0.01). EIH was associated with a nearly 2-fold increase in the risk of cardiovascular events (hazard ratio, 1.97 [95% CI, 1.52–2.43]; *P*=0.009; Table 4). The addition of exercise SBP to the base model resulted in improvement in predictive accuracy as evidence by an increase in *C*-statistic from 0.671 (0.645–0.694) to 0.727 (0.709–0.750); *P*=0.01, Table 5. However, the addition of exercise DBP to the base model did not result in significant improvement in predictive accuracy (from 0.671 [0.645–0.694] to 0.689 [0.652–0.706]; *P*=0.4). Similarly, the addition of exercise PP to the base model did not result in significant improvement in predictive accuracy (from 0.671 [0.645–0.694] to 0.696 [0.655–0.712]; *P*=0.2, *P*=0.1).

**Table 4.**
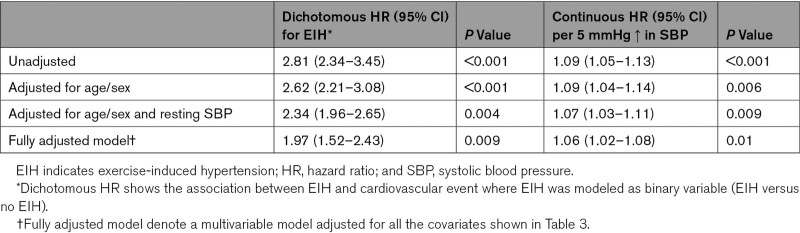
Cox Model Showing Association Between Exercise Systolic Blood Pressure and Cardiovascular Events

**Table 5.**
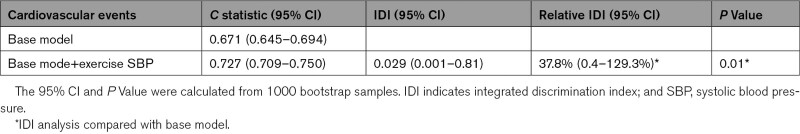
Discrimination Analyses for Prognostic Value of Exercise SBP

### Sensitivity Analyses

We performed 6 sets of pre-specified sensitivity analyses, and in these analyses, exercise SBP was associated with cardiovascular events in men, women, patients with isolated rCOA, patients with concomitant LV outflow disease, and patients without intensification of antihypertensive therapy (Table S2). However, there was no significant association between exercise SBP and cardiovascular events in patients that received intensification of antihypertensive therapy after exercise test (Table S2).

## Discussion

In this study, we assessed the prognostic role of exercise BP in adults with rCOA and treated hypertension. The main findings were (1) EIH was present in 35% of the cohort, and the patients with EIH had higher arterial load and more advanced LV remodeling as compared with the patients without EIH although both groups had similar resting BP. (2) Exercise SBP (whether modeled as continuous or binary variable) was associated with cardiovascular events independent of BP at rest. (3) The patients with EIH were less likely to be on ACEI/ARB; (4) The use of ACEI/ARB was associated with a lower risk of cardiovascular events.

Hypertension is common in adults with rCOA, and it is an independent risk factor for adverse outcomes.^1–4^ SBP measured at rest is the most common BP metric used for the diagnosis and monitoring of hypertension, but it tends to underestimate LV pressure load, and in turn, leads to under-diagnosis and potentially under-treatment of hypertension in patients with rCOA.^8–10^ As a result, the American and European guidelines for the management of adults with congenital heart disease endorse the use of alternate BP measurement modalities such as ambulatory BP monitoring and BP assessment during exercise.^5–7^ EIH in patients with normal BP at rest is associated with a higher risk for developing chronic hypertension during follow-up, and hence can be used for screening and early diagnosis of hypertension.^17–23^ The current study supports a novel application of exercise BP for prognostication and for assessment of adequacy of antihypertensive therapy in adults with rCOA. These findings are consistent with studies conducted in the acquired heart disease population, showing that EIH was a risk factor for cardiovascular mortality, independent of office BP and other atherosclerotic cardiovascular disease risk factors.^28,29^

We also observed that patients with EIH had higher Doppler derived arterial load indices (higher effective arterial elastance index and lower total arterial compliance index) as compared with patients without EIH, although both groups had similar resting BP. This is consistent with previous studies suggesting that SBP measured at rest may not provide an accurate assessment of LV afterload in the rCOA population.^10,30,31^ This discordance between resting SBP and central aortic pressure has been attributed to factors such as difference in aortic stiffness, wave reflection from impedance mismatch of the thoracic aorta, and sympathoadrenergic response during activity. We postulate that the EIH group had higher arterial load (that was not apparent from resting SBP data alone), and this in turn, led to more advanced LV remodeling (LV hypertrophy, systolic and diastolic dysfunction), and worse outcomes as compared with patients without EIH.

## Clinical Implications

The improved prognostic power of exercise SBP (as compared to resting SBP alone) in the current study, suggests that exercise SBP can be used to assess the adequacy of antihypertensive therapy in rCOA patients with hypertension. For instance, 29% of the 146 patients with optimal SBP control had EIH and higher incidence of cardiovascular events (Figure [B]), and perhaps, these patients would benefit from more intensive antihypertensive therapy. Of note, exercise SBP was associated with cardiovascular events in all subgroup analyses except in the subset of patients that had intensification of antihypertensive therapy following exercise test demonstrating. This suggests that titration of antihypertensive therapy using exercise BP may improve outcomes in this population.

Furthermore, we observed that the patients with EIH were less likely to be on ACEI/ARB, ant that the use of ACEI/ARB was associated with a lower risk of cardiovascular events as compared with other antihypertensive medications. We postulated that this may be related to the role of angiotensin II in the pathogenesis of EIH and adverse LV remodeling (increased LV hypertrophy and stiffness, and impaired LV relaxation).^32,33^ Angiotensin II is elevated in patients with hypertension, and the highest angiotensin II levels occurred during exercise in patients with EIH.^32^ ACEI/ARB has been shown to decrease BP at peak exercise and improve LV diastolic function, without any change in resting BP, further supporting the important role of neurohormonal activation in the pathogenesis of EIH.^33,34^ However, similar data are lacking in patients with rCOA.

## Limitations

This is a retrospective, observational single-center study and hence it is prone to selection and ascertainment bias, and inferences on causality cannot be drawn. The resting BP used in this study was derived from office BP (BP measured in the clinical setting), and the office BP is the least reliable method for assessing hypertension. However, we tried to mitigate this limitation by using BP measured in 3 different clinical settings and by 3 different trained operators, and this assessment mirrors what is often obtained in everyday clinical practice. Furthermore, we did not measure biomarkers of neurohormonal activation and cardiac remodeling, and hence are unable to provide mechanistic insight into the underlying pathophysiologic interactions between exercise BP, LV remodeling, and the effect of neurohormonal blockade. The association between ACEI/ARB use is prone to confounding due to indication and other measured and unmeasured group differences and must be considered as hypothesis-generating only. However, the present data emphasize the importance of performing adequately powered randomized controlled trials to determine whether treatments to reduce EIH might improve outcomes in patients with rCOA.

## Conclusions

EIH was present in 35% of adult with rCOA treated hypertension and was commonly observed even in the subset of patients with normal BP at rest. EIH was associated with cardiovascular events independent of BP at rest. The patients receiving ACEI/ARB were less likely to have EIH, and also had a lower risk of cardiovascular events. These data suggest that exercise BP can potentially be used to assess adequacy of antihypertensive therapy, and in turn, to guide the titration of antihypertensive therapy. Further studies are required to determine whether the integration of exercise BP into the clinical decision process would improve clinical outcomes in adults with rCOA.

### Perspectives

The current study assessed the relationship between EIH and cardiovascular events, and whether exercise BP improved risk stratification in adults with rCOA. The result showed that EIH was present in 35% of adult with rCOA treated hypertension and was commonly observed even in the subset of patients with normal BP at rest. EIH was associated with cardiovascular events independent of BP at rest. The patients receiving ACEI/ARB were less likely to have EIH and also had a lower risk of cardiovascular events. These data suggest that exercise BP can potentially be used to assess adequacy of antihypertensive therapy, and in turn, to guide the titration of antihypertensive therapy. Further studies are required to determine whether the integration of exercise BP into the clinical decision process would improve clinical outcomes in adults with rCOA.

## Article Information

### Sources of Funding

A.C. Egbe is supported by National Heart, Lung, and Blood Institute (NHLBI) grants (K23 HL141448, R01 HL158517 and R01 HL160761). The MACHD Registry is supported by the Al-Bahar Research grant. B.A. Borlaug is supported by research grants for the NHLBI (R01 HL128526 and U01 HL160226).

### Disclosures

None.

## Supplementary Material


