# Supplementary material for: Prognostic Implications of Exercise-Induced Hypertension in Adults With Repaired Coarctation of Aorta
Source: Hypertension. 2022 Oct 12;79(12):2796–805. doi: 10.1161/HYPERTENSIONAHA.122.19735 (PMC9640261; doi:10.1161/HYPERTENSIONAHA.122.19735)
Supplement: Supplementary file 1 [file hyp-79-2796-s001.pdf]

Prognostic Implications of Exercise Induced Hypertension in Adults with Repaired Coarctation of Aorta

Alexander C. Egbe MD, MPH; William R. Miranda, MD; C. Charles Jain, MD; Barry A. Borlaug, MD; Heidi M. Connolly, MD

From the Department of Cardiovascular Medicine, Mayo Clinic Rochester, MN 55905

Short Title: Hypertension in Coarctation of Aorta

Word Count: Abstract 250; Text 2,994

Conflict of Interest: none

Disclosures: none

Address for correspondence:
Alexander Egbe, MD MPH, FACC
Mayo Clinic and Foundation
200 First Street SW
Rochester, MN 55905
Phone: 507-284-2520
Fax: 507-266-0103
Email: egbe.alexander@mayo.edu

Supplementary Table S1: Baseline Characteristics

	All (n=327)	Isolated rCOA (n=215, 66%)	rCOA with LVOD (n=112, 34%)	p
Age, years	35±13	36±10	33±9	0.1
Male	201 (62%)	131 (61%)	70 (63%)	0.4
Age of COA repair, years	3 (1-6)	3 (2-6)	1 (1-3)	0.1
NYHA III/IV	44 (13%)	18 (8%)	26 (23%)	<0.001
ULE SBP gradient, mmHg	12 (8-15)	13 (7-16)	10 (6-15)	0.6
**Comorbidities**				
Coronary artery disease	16 (5%)	11 (5%)	5 (5%)	0.9
Diabetes	15 (5%)	12 (6%)	3 (6%)	0.9
Atrial fibrillation	28 (9%)	12 (6%)	16 (14%)	0.008
**Medications**				
ACEI/ARB	192 (59%)	128 (60%)	64 (57%)	0.2
Aldosterone antagonist	8 (2%)	4 (2%)	4 (4%)	0.8
Beta blockers	168 (51%)	97 (45%)	71 (63%)	0.009
Calcium channel blockers	38 (12%)	24 (11%)	14 (13%)	0.7
Thiazide diuretics	33 (10%)	21 (10%)	12 (11%)	0.8
Others	13 (4%)	8 (4%)	5 (3%)	0.9
**Laboratory data**				
Estimated GFR, ml/min/1.73m^2	90±21	96±19	84±16	0.6
**Echocardiographic data**				
Aortic mean gradient, mmHg	14±8	11±4	26±5	<0.001
≥ Moderate Aortic regurgitation	19 (6%)	0	19 (17%)	---
COA mean gradient, mmHg	13±4	13±4	12±4	0.4
Septal E/e’	12.1±5.7	11.2±3.1	13.9±3.4	0.005
LV mass index, g/m^2	104±19	94±18	122±21	<0.001
LV ejection fraction, %	62±8	64±10	58±8	0.6
LVGLS, %	-21±3	-22±3	-18±4	0.004
RVGLS, %	-24±4	-25±4	-22±3	0.09
RV fractional area change, %	45±8	46±9	43±7	0.4
RV systolic pressure, mmHg	34±12	32±10	39±11	0.08
**Average resting BP**				
SBP, mmHg	129±18	127±20	132±16	0.6
DBP, mmHg	72±12	71±11	74±10	0.8
PP, mmHg	57±15	56±12	58±13	0.9
**Doppler-derived arterial load indices**				

EAI, mmHg/ml*m^2	3.3±0.6	3.2±0.5	3.4±0.4	0.9
TACI, mL/mmHg*m^2	0.8±0.4	0.7±0.3	0.8±0.4	0.8
**Exercise data**				
SBP at peak exercise, mmHg	173±31	176±27	165±29	0.3
HR at peak exercise, bpm	164±28	168±36	161±27	0.2
RER	1.16±0.04	1.15±0.05	1.17±0.08	0.3
Exercise time, minutes	7.6±1.2	7.8±1.0	7.3±0.8	0.1
Peak VO_2, ml/kg/min	26±6	28±6	23±5	0.03

LVOD: left ventricular outflow disease; NYHA: New York Heart Association; ACEI: angiotensin converting enzyme inhibitor; ARB: angiotensin receptor blocker; coarctation of aorta; GFR: glomerular filtration rate; E/e’: ratio of mitral inflow pulsed wave early velocity to tissue Doppler early velocity; LV: left ventricle; RV: right ventricle; GLS: global longitudinal strain; ULE: upper to lower extremity; SBP: systolic blood pressure; DBP: diastolic blood pressure; PP: pulse pressure; HR: heart rate; RER: respiratory exchange ratio; VO_2: oxygen consumption; EAI: Effective arterial elastance index; TACI: Total arterial compliance index

Supplemental Table S2: Multivariable Cox Regression Models for Sensitivity Analyses

	HR (95% CI) for 5 mmHg increase in exercise					
	**Model A**	**Model B**	**Model C**	**Model D**	**Model E**	**Model F**
**Unadjusted**	1.08 (1.04-1.12)	1.07 (1.04-1.10)	1.08 (1.05-1.11)	1.06 (1.03-1.09)	1.04 (1.01-1.07)	1.07 (1.04-1.10)
**Adjusted***	1.06 (1.03-1.09)	1.05 (1.02-1.08)	1.06 (1.03-1.09)	1.04 (1.01-1.07)	1.02 (0.98-1.04)	1.05 (1.02-1.08)
	**HR (95% CI) for EIH versus no EIH**					
**Unadjusted**	2.74 (2.41-3.06)	2.90 (2.53-3.45)	2.69 (2.27-3.03)	2.44 (1.96-2.98)	1.83 (1.19-2.57)	2.91 (2.37-3.41)
**Adjusted ***	2.04 (1.65-2.46)	2.14 (1.71-2.66)	1.96 (1.61-2.37)	2.11 (1.28-3.07)	1.65 (0.85-2.46)	2.52 (2.01-3.03)

HR: hazard ratio; CI: confidence interval; Adjusted* signified HR adjusted for age and resting SBP.

Model A: Analysis restricted to males (n=201)

Model B: Analysis restricted to females (n=126)

Model C: Analysis restricted to patients isolated COA (n=215). Isolated COA was defined as the absence of concomitant LV outflow disease defined as having any of the following conditions: aortic valve prosthesis, sub-valvular, valvular, or supra-valvular aortic stenosis (mean gradient >20 mmHg) or ≥moderate aortic regurgitation.

Model D: Analysis restricted to patients with LV outflow disease (n=112)

Model E: Analysis restricted to patients that had intensification of antihypertensive therapy after exercise test (n=89)

Model F: Analysis restricted to patients without intensification of antihypertensive therapy after exercise test (n=239)

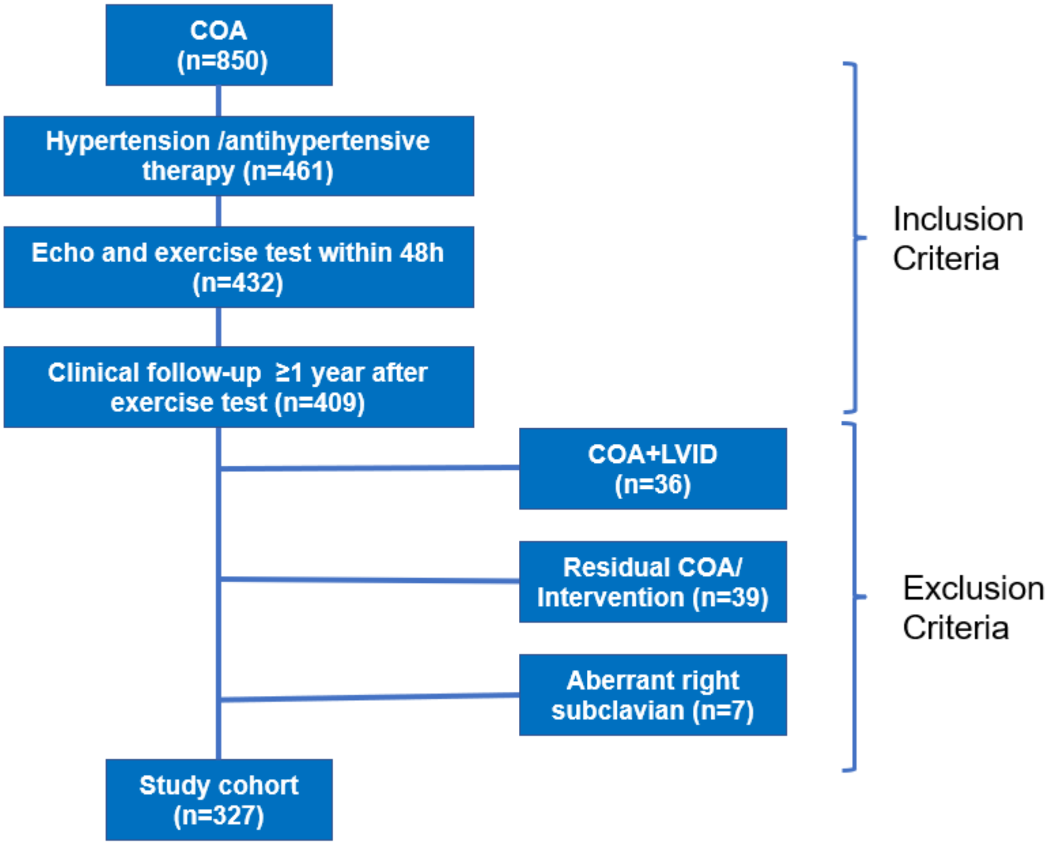

Supplementary Figure S1: A flowchart showing patient selection for the study.
COA: coarctation of aortal LVID: left ventricular inflow disease
